# Gandouling ameliorates Wilson’s disease-associated liver fibrosis in mice with associated faecal microbiome and metabolome remodeling

**DOI:** 10.3389/fphar.2025.1586171

**Published:** 2025-06-09

**Authors:** Lulu Tang, Wei Dong, Danqing Liu, Chenling Zhao, Jie Chen, Yuya Wen, Jingyu Zeng, Ting Dong, Wenming Yang

**Affiliations:** ^1^ Department of Neurology, The First Affiliated Hospital of Anhui University of Chinese Medicine, Hefei, Anhui, China; ^2^ Department of Neurology, Wuhan Hospital of Traditonal Chinese Medicine, Wuhan, Hubei, China; ^3^ Department of Medical Technology, Clinical College of Anhui Medical University, Hefei, Anhui, China; ^4^ Key Laboratory of Xin'an Medicine Ministry of Education, Hefei, Anhui, China

**Keywords:** Gandouling, Wilson’s disease, liver fibrosis, toxic milk mice, gut microbiota

## Abstract

**Introduction:**

Individuals with Wilson's disease (WD) exhibit liver fibrosis, a basic pathological change that was recently demonstrated to be dynamic and reversible. The gut microbiota markedly influences the occurrence of WD. Gandouling (GDL), a standardized Chinese herbal formula, has demonstrated an anti-fibrotic effect against WD-associated liver fibrosis. We sought to determine whether GDL may prevent liver fibrosis in toxic milk (TX) mice by assessing its ability to regulate gut microbiota, metabolites, and barrier function.

**Methods:**

TX male mice aged 6 months were analysed. GDL was administered at varying doses over a 6-week period. The biochemical indexes related to liver function, fibrosis, and inflammation were determined using commercial assay kits. Histological analyses and immunohistochemistry staining, were performed to evaluate the histopathological changes and collagen deposition in mouse liver tissues. Additionally, to detect alterations in the intestinal bacterial composition and metabolites, faecal samples were examined using non-targeted metabolomics and 16S rRNA sequencing.

**Results:**

The administration of GDL demonstrated anti-fibrotic effects on the liver, decreased serum inflammatory markers, ameliorated liver histopathology, and restored ileal permeability in the model group, as compared to the control group. Furthermore, a medium dosage of GDL treatment significantly rebalance microbiota composition and function and modulated lipid and lipid-like molecule levels.

**Discussion:**

Modulating intestinal homeostasis is a promising approach for treating liver fibrosis in patients with WD. Therefore, GDL may serve as a useful agent for treating WD-associated liver fibrosis.

## Graphical Abstract

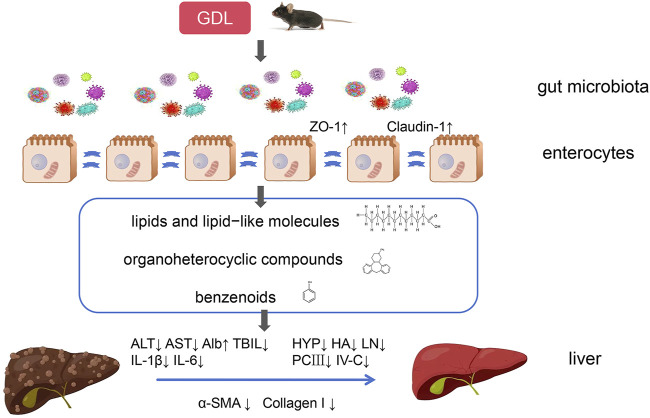

## Introduction

Wilson’s disease (WD) is an autosomal recessive genetic disorder caused by *ATP7B* mutations that induce impaired copper metabolism. China has a much higher percentage of patients with WD than that of Western countries, with the global incidence of WD being approximately 1 in 30,000 individuals (Xie and Wu, 2017). WD causes organ damage, particularly in the liver, and induces neurological impairment. The initial pathology associated with early-stage WD hepatic disease is steatosis, which progresses to liver fibrosis, cirrhosis, and mortality. One of the primary pathological changes in patients with WD is liver fibrosis, which has recently been confirmed to be dynamic and reversible (Gerosa et al., 2019).

Earlier research on animals has demonstrated that copper has a direct impact on the composition of gut bacteria (Pajarillo et al., 2021). Similarly, altered gut microbial environments suggest that the metabolic dysfunction in WD may inherently alter gut microbiota, promoting inflammation and exacerbating the underlying disease (Geng et al., 2018; Cai et al., 2020). The gut microbiota is crucial in the occurrence of WD; therefore, the regulation of gut microbiota is potentially a novel strategy for WD treatment.

The gut microbiota plays a key role in maintaining normal human physiological processes, and its ecological imbalance can contribute to liver disease and influence the disease’s progression (Rager and Zeng, 2023). When the integrity of the intestinal barrier is compromised, the advancement of liver fibrosis may be significantly accelerated. When this barrier is disrupted, pathogen-associated molecular patterns (PAMPs), such as abnormal bacterial fragments, endotoxins, and other metabolites, can enter the portal venous system and reach the liver. The immunological responses triggered by these PAMPs entering the liver can lead to the release of pro-inflammatory cytokines, which exacerbate liver fibrosis (Zhou et al., 2019). Traditional Chinese medicines (TCMs) have emerged as distinctive agents for WD treatment because of their multi-targeted, phased, and multi-faceted treatment characteristics (Xu et al., 2019). Gandouling (GDL), a standardized Chinese herbal formula, has demonstrated an anti-fibrotic effect against WD-associated liver fibrosis (Approval No. Z20050071). Empirical evidence indicates that GDL facilitates the elimination of accumulated copper through renal metabolic pathways and helps reconstruct the biliary tract to expel copper from bodily tissues (Zhang et al., 2018a; Zhang et al., 2018b). GDL primarily comprises *Rheum officinale* Baill [Polygonaceae; *Rhei Radix et Rhizoma*], *Coptis chinensis* Franch [Ranunculaceae; *Coptidis Rhizoma*], *Salvia miltiorrhiza* Bunge [Lamiaceae; *Salviae Miltiorrhizae Radix et Rhizoma*], *Spatholobus suberectus* Dunn [Fabaceae; *Caulis Spatholobi*], *Curcuma longa* L [Zingiberaceae; *Curcumae Longae Rhizoma*] and *Curcuma phaeocaulis* Valeton [Zingiberaceae; *Rhizoma Curcumae Phaeocaulis*] in the ratios of 12:27:14:14:12:12. The GDL preparation procedure was optimized through the use of orthogonal design (Lv, 1999). HPLC was used to monitor the concentration of GDL’s active ingredients for quality control purposes (Li et al., 2020). Ultra-performance liquid chromatography coupled with quadrupole time-of-flight mass spectrometry (UPLC-Q-TOF-MS) was employed to comprehensively characterize the chemical constituents of GDL, including its prototype phytochemicals and metabolites, in plasma, liver, and urine samples collected from GDL-treated rats (Wang et al., 2025). The 2023 “Guidelines for the Diagnosis and Treatment of Wilson’s Disease with Integrated Traditional and Biomedicine Medicine” in China recommend the use of GDL for the treatment of patients with WD-mediated liver injury (Cheng et al., 2023; Zhao et al., 2024).

The main objective of this study is to determine whether GDL can ameliorate liver fibrosis in toxic milk mice (TX) and clarify the protective mechanism of GDL against liver fibrosis by non-targeted metabolomics and *16S* rRNA sequencing.

## Materials and methods

### Animals and treatments administered

The Jackson Laboratory’s Experimental Animal Center (Bar Harbor, ME, United States) provided the six-month-old male specific pathogen-free (SPF) toxic milk (TX) mice (strain: C3HeB/FeJAtp7btx-J/J), which were raised at the College of Life Sciences, Anhui Agricultural University, on a standard diet and under SPF conditions. Each mouse weighed 20–35 g and were housed under the following strictly controlled conditions: temperatures of 18–20°C, a defined light-dark cycle, humidity levels of 50%–60%, and unrestricted access to food and water. We randomly assigned 24 pure TX mice into four groups: model and GDL (high-dose, medium-dose, and low-dose) groups. Through gavage, the mice in the GDL group were administered with GDL tablets (GDL high-dose: 1.16 g/kg/day; GDL medium-dose: 0.58 g/kg/day; and GDL low-dose: 0.29 g/kg/day) manufactured by the First Affiliated Hospital of Anhui University of Chinese Medicine (Nair and Jacob, 2016; Tian et al., 2024; Wen et al., 2024) (Batch No. 20220618). Furthermore, the control group consisted of six wild-type mice with identical genetic backgrounds. Equal volumes of distilled water were administered by gavage to the model and control groups. The Anhui University of Chinese Medicine Ethics Committee granted ethical approval for the experiments (approval number: AHUCM-mouse-2021011). After a 12-h fast without any food or drink following 6 weeks of treatment, all mice were anesthetized, and blood samples were taken from their eyes. The liver and ileum tissues were immediately extracted and stored in 4% paraformaldehyde for further analysis.

### Liver organ indices

The mice were weighed before they were sacrificed. After extracting the liver tissues, they were rinsed with a 0.9% saline solution and blotted. The liver coefficient was determined using the following method:

$$\text{Liver coefficient }\left(\%\right)=\text{liver weight}/\text{total body weight}$$

### Serum biochemical analysis

Albumin (ALB), total bilirubin (TBIL), alanine aminotransferase (ALT), aspartate aminotransferase (AST), interleukin-6 (IL-6), interleukin-1β (IL-1β), hydroxyproline (HYP), hyaluronic acid (HA), laminin (LN), precollagen type III (PCIII), and collagen IV (IV-C) levels were measured using ELISA kits. Each biochemical test adhered to the instructions given by the manufacturer.

### Histopathological and immunohistochemical examinations

Fresh liver and intestinal tissues were preserved in 4% paraformaldehyde and cut into 5 μm thick sections. The liver samples were then stained with hematoxylin-eosin (HE), Masson and Sirius Red to assess inflammation and fibrosis. Microscopic images were analyzed using ImageJ 1.8.0 for the Masson and Sirius Red stains. Additionally, liver sections were treated with antibodies for collagen I and alpha-smooth muscle actin (α-SMA), while paraffin-embedded ileum sections were stained with antibodies for zonula occludens-1 (ZO-1) and claudin-1.

### *16S* rRNA gene sequencing and gut microbiota analysis

Total DNA from mouse faeces collected from the control (n = 6), model (n = 6), and GDL (medium-dose group, n = 6) groups was extracted using the cetyltrimethylammonium bromide (CTAB) method. Before performing a polymerase chain reaction (PCR), the complete DNA was stored at −80°C following its elution in 50 μL of an elution buffer. Primers were used to target the amplification of the V3-V4 regions of the 16S rRNA gene. Using AMPure XT beads from Beckman Coulter Genomics in Danvers, MA, United States, the PCR products were purified and then quantified with Invitrogen’s Qubit, United States. The size and quantity of the amplicon library were measured using an Agilent 2100 Bioanalyzer (Agilent, United States) and the Library Quantification Kit for Illumina platforms (Kapa Biosciences, Woburn, MA, United States). Libraries were sequenced using a NovaSeq PE250 platform. Data preprocessing involved the utilization of FLASH for raw paired-end reads assembly and quality control. This process yielded clean tags, which were subsequently analyzed using QIIME2. Alpha diversity, including Chao1, Observed species, Goods coverage, Shannon, Simpson, and all this indices in our samples were calculated. Beta diversity was measured using analysis of similarities (ANOSIM) and principal coordinate analysis (PCoA). To identify significantly abundant bacterial taxa among the different groups, the linear discriminant analysis (LDA) effect size was assessed using the LEfSe online tool.

### Untargeted metabolomic analysis

After collecting 25 mg of fecal matter, 500 μL of an extraction solution containing deuterated internal standards (methanol, acetonitrile, and water in a 2:2:1 volume ratio) was added, and the mixture was vortexed for 30 s. The samples were then homogenized for 4 min at a frequency of 35 Hz and sonicated for 5 min in a water bath that was kept at 4°C, three times. To aid in protein precipitation, the samples were incubated at −40°C for 1 h, and then centrifuged for 15 min at a speed of 12,000 rpm, which corresponded a relative centrifugal force of 13,800 × g with a rotor radius of 8.6 cm. Liquid chromatography-tandem mass spectrometry (LC-MS/MS) studies were performed using a Vanquish UHPLC system (Thermo Fisher Scientific) for the study of polar metabolites. Information-dependent acquisition mode was used to get MS/MS spectra using the Orbitrap Exploris 120 mass spectrometer. ProteoWizard was used to convert the raw data to the mzXML format, and an internal R-based application that used XCMS was used to handle the data. Raw peaks were filtered by relative standard deviation (RSD) denoising. Missing values were imputed with half the minimum value, followed by total ion current (TIC) normalization. The processed dataset (peak number, sample name, normalized peak area) was analyzed using SIMCA 16.0.2. Principal component analysis (PCA) visualized sample distribution and identified outliers. Orthogonal partial least squares-discriminant analysis (OPLS-DA) was applied for group separation. Metabolites with VIP >1 and *p* < 0.05 were deemed significant. Pathway enrichment analysis utilized KEGG and MetaboAnalyst databases.

### Statistical analysis

The data from independent experiments were presented as the mean ± standard deviation. For comparing multiple groups, a one-way ANOVA was conducted, followed by Fisher’s least significant difference post-hoc test using GraphPad Prism (9.0.0). For repeated measures data, a two-way ANOVA was performed with two independent variables. Tukey-Kramer’s multiple comparisons test was used for post-hoc analyses. Statistical significance was set at *p* < 0.05.

## Results

### GDL ameliorates liver damage in TX mice

**FIGURE 1:**
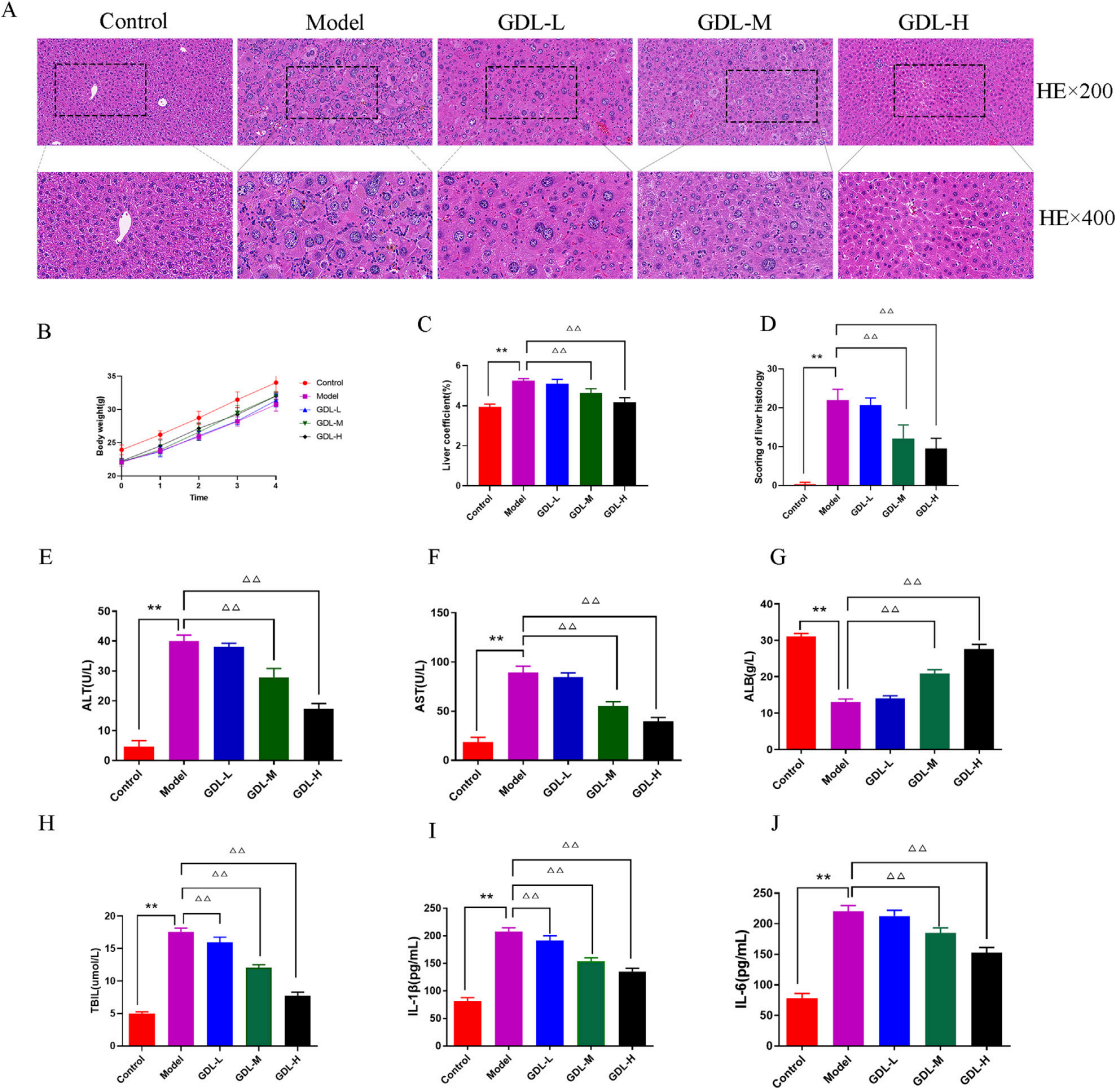
Beneficial effects of different doses of GDL administration against liver damage. **(A)** Histological representations of the liver (magnification ×400, scale bar = 20 μm, n = 3), **(B)** Body weight (the trend of body weight), **(C)** Liver coefficient, **(D)** Scoring of liver histology, and **(E–J)** Serum levels of aminotransferase (ALT), aspartate aminotransferase (AST), albumin (ALB), total bilirubin (TBIL), interleukin (IL)-1β, and IL-6. Data were expressed as the mean ± standard deviation (SD) (n = 6). **p < 0.01, against the control group; ^△△^ *p* < 0.01, against the model group.

The body weights of the model group mice were lower than those of the control group at baseline (Figure 1B). After administering GDL, there were no notable changes in body weight across the various weeks. The liver coefficient was higher in the model group compared to the control group, but it decreased following GDL administration (1.16 and 0.58 g/kg; all, *p* < 0.01; Figure 1C). The normal control group mice hepatocytes were closely packed, according to HE staining, and there were no visible inflammatory cell infiltration or hepatic parenchymal cell abnormalities. The model group mice showed significant collagen fibre proliferation, eosinophilic cytoplasmic enhancement, and hepatocyte steatosis. Additionally, a minor degree of lymphocyte infiltration was noted in the lobule portal regions. To differing degrees, GDL treatments (1.16 and 0.58 g/kg) attenuated the pathological alterations in the liver tissues (Figures 1A,D). The serum biochemical indices are displayed in Figures 1E–H. The model group had significantly higher ALT, AST, and TBIL levels, and lower ALB levels than those of the control group (all, *p* < 0.01). Compared with those of the model group, the GDL groups (1.16 and 0.58 g/kg) showed significantly decreased ALT, AST, and TBIL levels and increased ALB levels (all, *p* < 0.01). Additionally, the model mice showed increased serum IL-6 and IL-1β levels, which were significantly decreased after GDL intervention (Figure IH and J; all *p <* 0.01).

### GDL ameliorates liver fibrosis in TX mice

**FIGURE 2:**
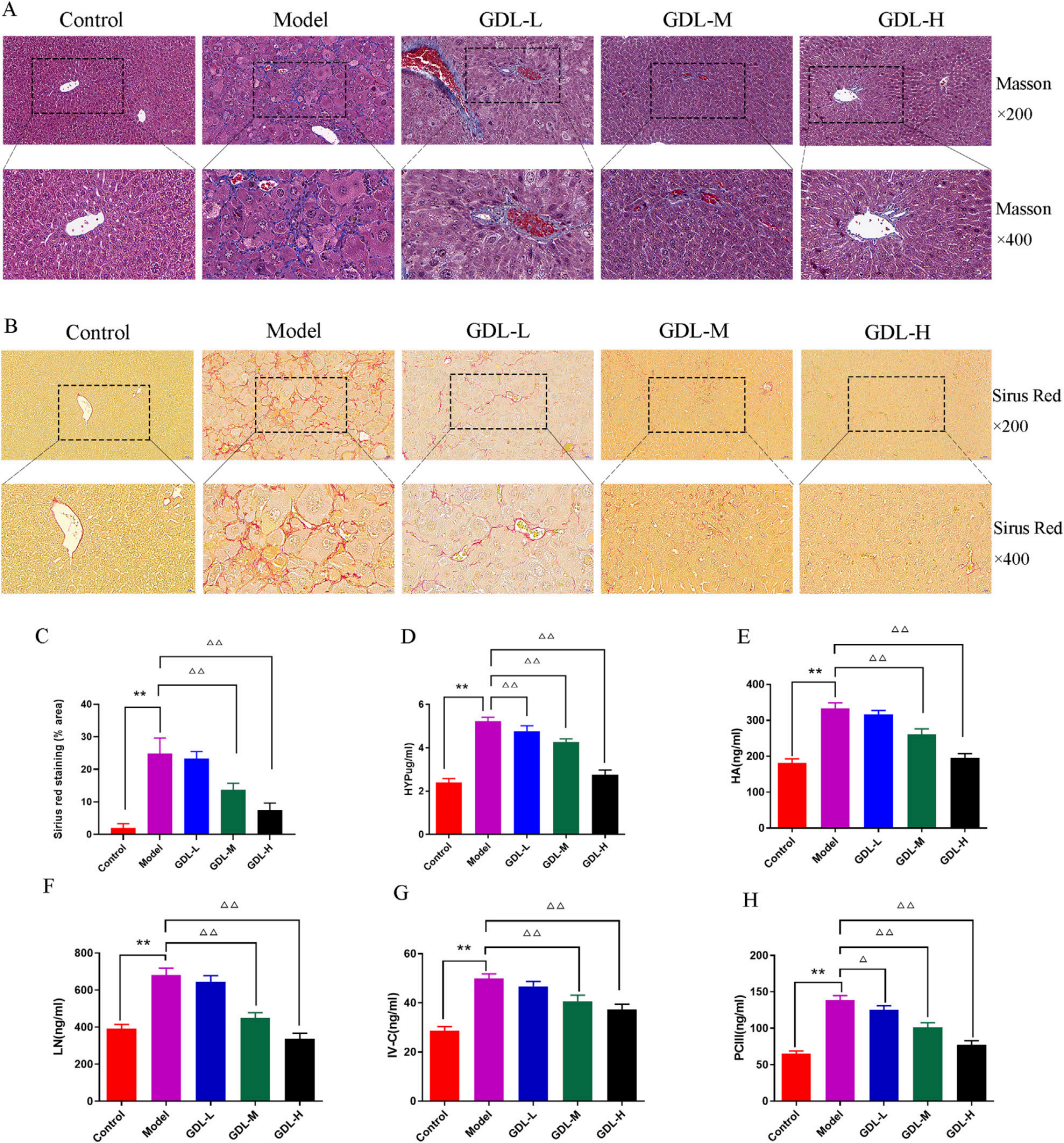
Beneficial effects of different doses of GDL administration against liver fibrosis. **(A)** Masson staining (magnification ×400, scale bar = 20 μm, n = 3), **(B)** Sirius red staining (magnification ×400, scale bar = 20 μm, n = 3), **(C)** Sirius red staining (% area), and **(D–H)** Serum levels of hydroxyproline (HYP), hyaluronic acid (HA), laminin (LN), precollagen Ⅲ (PCⅢ), and Collagen Ⅳ (IV-C). Data are expressed as the mean ± SD (n = 6). **p < 0.01 against the control group. ^△^ *p* < 0.05 and ^△△^ *p* < 0.01, against the model group.

The distribution of fibrous tissue was examined using Masson and Sirius Red staining. The collagen fibres were stained blue and red by Masson and Sirius Red stains, respectively (Figures 2A–C. According to the figure, the model mice produced a lot of collagen fibre, while the control group generated almost none. GDL (1.16 and 0.58 g/kg) significantly decreased the liver deposition of collagen. The liver fibrosis biochemical indices are presented in Figures 2D–H. The model group had significantly higher HYP, HA, LN, IV-C, and PCIII levels than those of the control group (all *p* < 0.01). Compared with those of the model group, the GDL groups (1.16, 0.58, and 0.29 g/kg treatments) had significantly lower HYP, HA, LN, IV-C, and PCⅢ levels (*p* < 0.05, *p* < 0.01).

**FIGURE 3:**
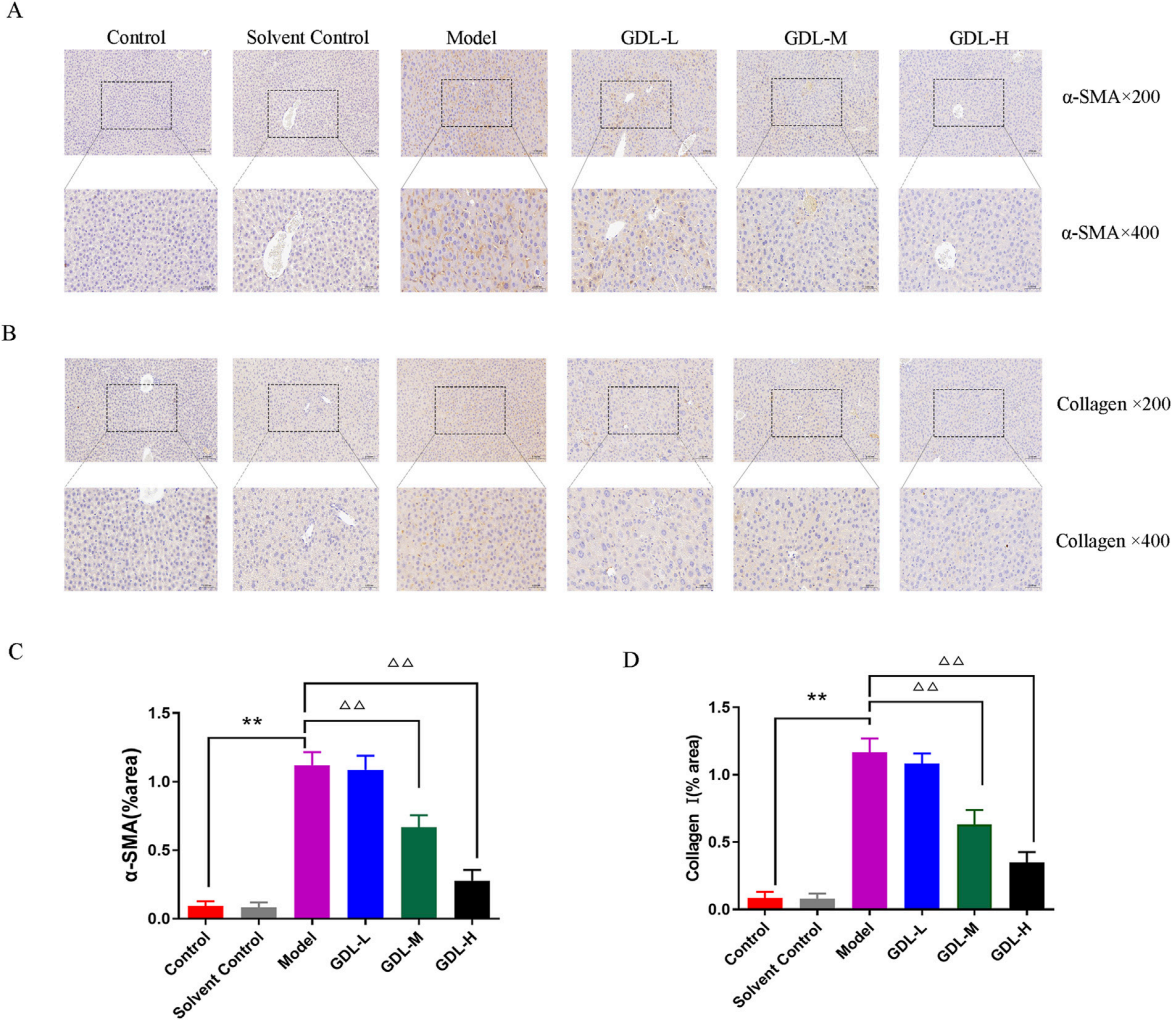
Beneficial effects of different dosages of GDL administration against liver fibrosis-associated hepatic stellate cell (HSC) activation. **(A,B)** Immunohistochemical staining of liver tissue for Collagen I and alpha-smooth muscle actin (α-SMA) (magnification ×400, scale bar = 20 μm, n = 3) and **(C,D)** Quantification of Collagen I and α-SMA in liver tissues. Data are expressed as the mean ± SD. **p < 0.01, against the control group, ^△△^ *p* < 0.01, against the model group.

The activation of hepatic stellate cells (HSCs) typically occurs in conjunction with liver fibrosis. The extracellular matrix (ECM) is primarily composed of collagen I and α-SMA is a sign of HSC activation. To investigate whether GDL prevents HSC activation against liver fibrosis, the expression levels of collagen I and α-SMA were taken into consideration. Collagen I and α-SMA exhibited dark staining (Figures 3A–D). The findings demonstrated that the model group had much more α-SMA- and collagen I-positive cells in their livers than those of the control group, which did not exhibit either of these proteins. In contrast, GDL (1.16 and 0.58 g/kg) treatments significantly reduced the numbers of α-SMA- and collagen I-immunoreactive cells (all *p* < 0.01).

### GDL modulates gut micro-ecology in TX mice

**FIGURE 4:**
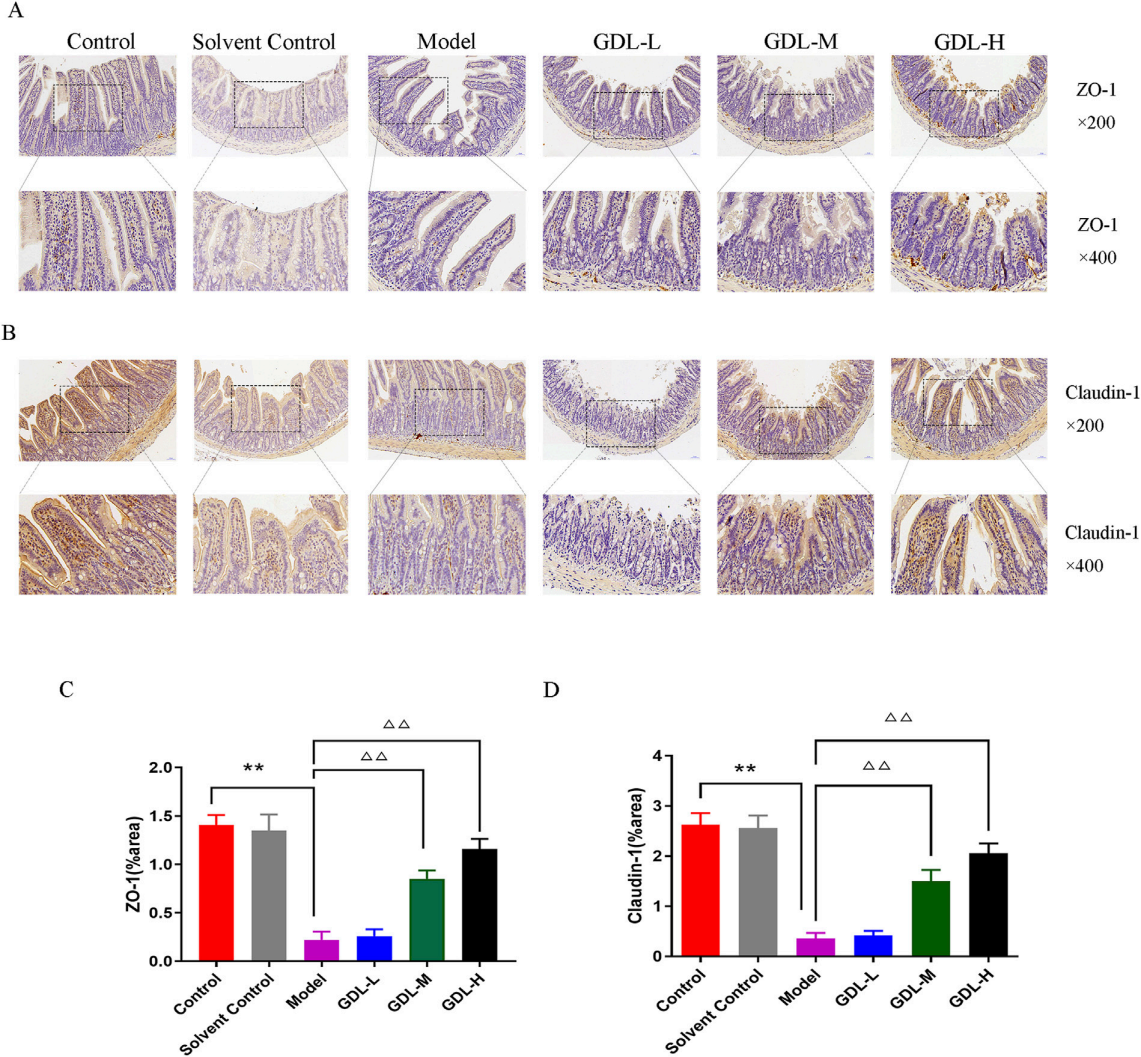
Beneficial effects of different doses of GDL administration on intestinal epithelial barrier function. **(A,B)** Immunohistochemical staining of intestinal tissues for ZO-1 and Claudin-1 (magnification ×400, scale bar = 20 μm, n = 3) and **(C,D)** Quantification of ZO-1 and Claudin-1 in intestinal tissues. Data are expressed as the mean ± SD. **p < 0.01, against the control group; ^△△^ *p* < 0.01, against the model group.

The expression levels of ileal ZO-1 and claudin-1 were dramatically reduced in the model mice (Figures 4A–D). GDL treatments (1.16 and 0.58 g/kg) restored ileal permeability and significantly increased ZO-1 and claudin-1 expression levels (all, *p* < 0.01).

**FIGURE 5:**
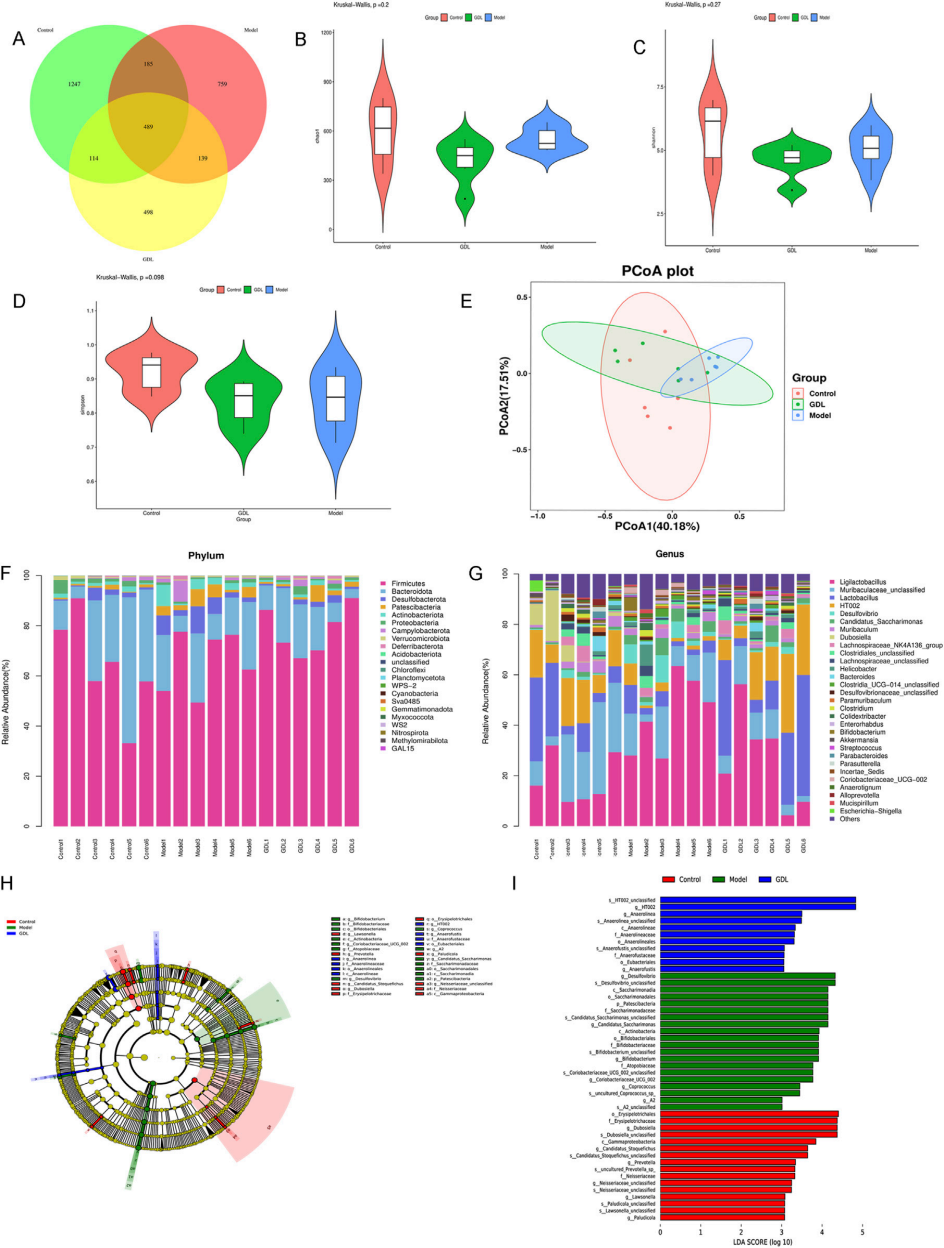
16S rRNA sequencing reveals alterations in the intestinal microbiota between toxic milk (TX) mice and middle-dose GDL treatment group. **(A)** Venn, **(B)** Chao, **(C)** Shannon, and **(D)** Simpson indices. Data are expressed as the mean ± SD. Statistical significance was assessed using Student’s t-test. **(E)** Principal coordinate analysis was performed based on Bray–Curtis dissimilarity to explore the gut microbiota profiles of the control (n = 6), model (n = 6), and GDL-M (n = 6) group mice. **(F,G)** Heatmap of the top 30 differentially abundant gut microbiota in the three groups at the phylum and genus levels. **(H,I)** LEfSe analysis indicates significantly enriched gut bacteria in the model and GDL-M groups.

Similarly, 16S rRNA sequencing was conducted to assess alterations in the gut microbiota of TX mice subsequent to medium-dose GDL administration. A total of 3,431 operational taxonomic units (OTUs) were detected in the faecal samples from the three groups (control, model, and medium-dose GDL). The Venn diagram shows that there are 489 shared OTUs and 1,247, 759, and 498 specific OTUs in the control, model, and GDL groups, respectively (Figure 5A). There were no significant differences in the Chao1, Shannon, or Simpson indices among the three groups, indicating that the alpha diversity of the gut microbiota community did not change in the TX mice after GDL intervention (Figures 5B–D). Principal coordinate analysis based on the Bray–Curtis distance and further ANOSIM analysis (R = 0.349383, *p* = 0.003) revealed significant dissimilarities among the three groups (Figure 5E). We conducted an analysis of the variations in the relative abundances of gut microbiota species. Our evaluation of the relative abundances of key bacterial taxa at both the phylum and genus levels revealed that the overall composition of the gut microbiota differed among the various groups (Figures 5F,G). At the phylum level, the control group exhibited significantly increased the abundances of Patescibacteria, Desulfobacterota, and Actinobacteriota, whereas GDL treatments decreased the abundance of Patescibacteria. The model group exhibited a decreased abundance of Bacteroidota; however, this change was not statistically significant (*p* > 0.05). At the genus level, there was a significant decrease in the relative abundances of *Olsenella*, *HT002*, *Burkholderia-Caballeronia-Paraburkholderia*, *Dubosiella, Faecalibaculum*, *Staphylococcus, Lactobacillus, Muribaculaceae_unclassified*, and *Alloprevotella* in the model group compared to those in the control group. The relative abundances of *Desulfovibrio, Candidatus_Saccharimonas, A2, Bifidobacterium, Coriobacteriaceae_UCG-002, Ligilactobacillus, Parasutterella, Mucispirillum*, and *[Eubacterium]_brachy_group* were significantly higher in the model group than those in the control group. GDL treatments significantly increased the relative abundance of *HT002* and decreased that of *Desulfovibrio, Coriobacteriaceae_UCG-002, A2*, and *Eubacterium]_brachy_group*. In addition, LEfSe analysis showed that the model group had relatively high abundances of *Desulfovibrio*, *Candidatus_Saccharimonas*, *Bifidobacterium*, *Coriobacteriaceae_UCG-002*, *Coprococcus,* and *A2* (at the genus level). In contrast, GDL primarily increased the abundances of *HT002*, *Anaerolinea,* and *Anaerofustis* (Figures 5H,I).

**FIGURE 6:**
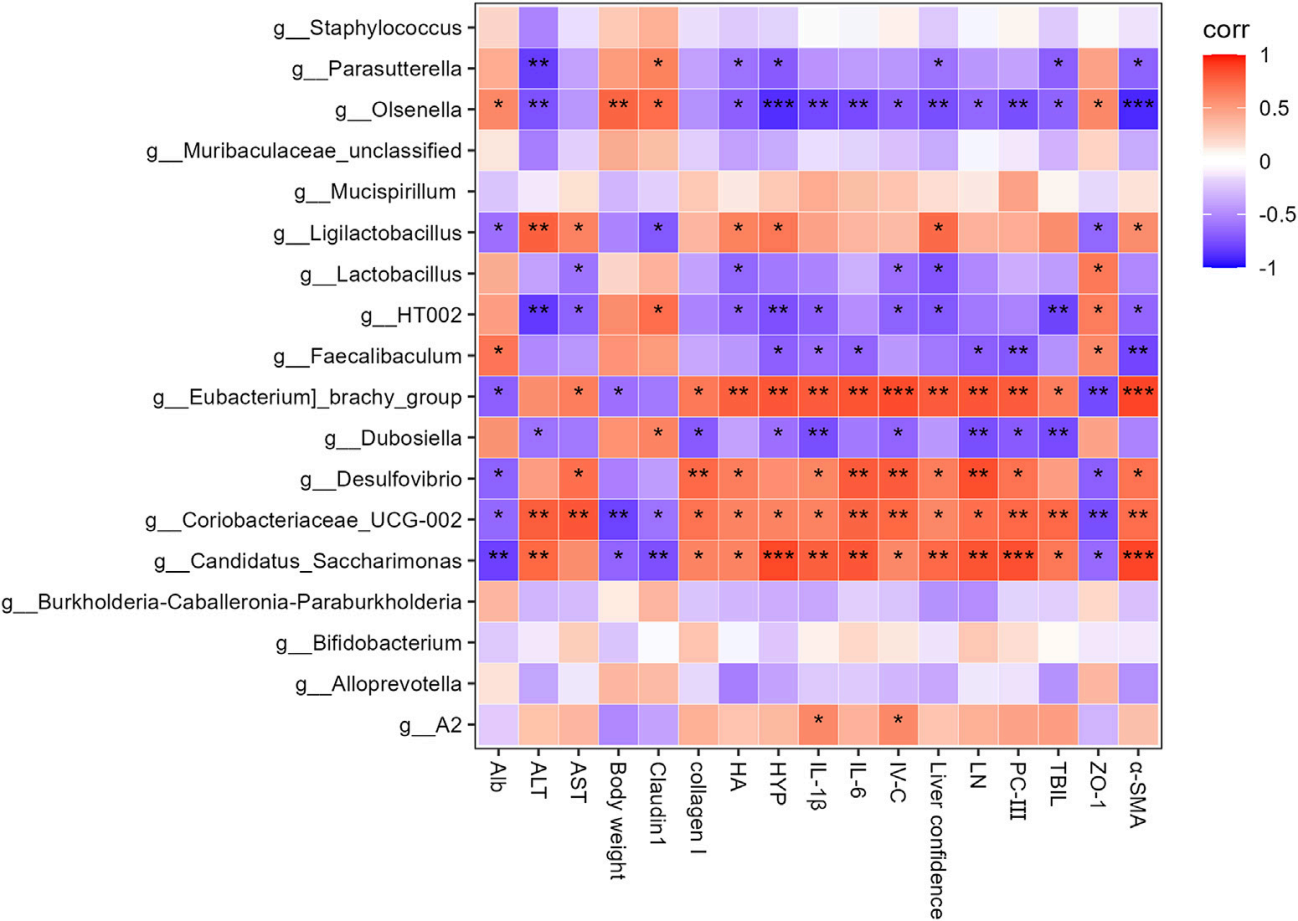
Heatmap of Spearman’s correlation coefficients between the Top 18 gut microbes (at the genus level) and liver function, liver fibrosis, inflammation, body weight, liver coefficient and intestinal barrier-related parameters. *p < 0.05, **p < 0.01, and ***p < 0.001.

Spearman’s correlation analysis evaluated the relationship between key gut bacterial genera and liver function, fibrosis, inflammation, and intestinal barrier integrity indices. *Candidatus_Saccharimonas, Coriobacteriaceae_UCG−002, Desulfovibrio*, and *[Eubacterium]_brachy_group* were positively correlated with liver function, fibrosis, coefficient, and inflammation, and negatively correlated with intestinal barrier integrity and ALB levels (Figure 6). *Dubosiella, Faecalibaculum, HT002*, and *Olsenella* were positively correlated with intestinal barrier integrity and negatively correlated with liver function, fibrosis, and inflammation. *Faecalibaculum* and *Olsenella* were positively correlated with ALB levels. *Olsenella* was positively correlated with the body weight index. *Ligilactobacillus* was positively correlated with liver function, fibrosis, and the coefficient, and negatively correlated with intestinal barrier integrity and ALB levels. *Lactobacillus* was positively correlated with intestinal barrier integrity and negatively correlated with liver function, fibrosis, and coefficient levels.

### GDL alters faecal metabolites in TX mice

**FIGURE 7:**
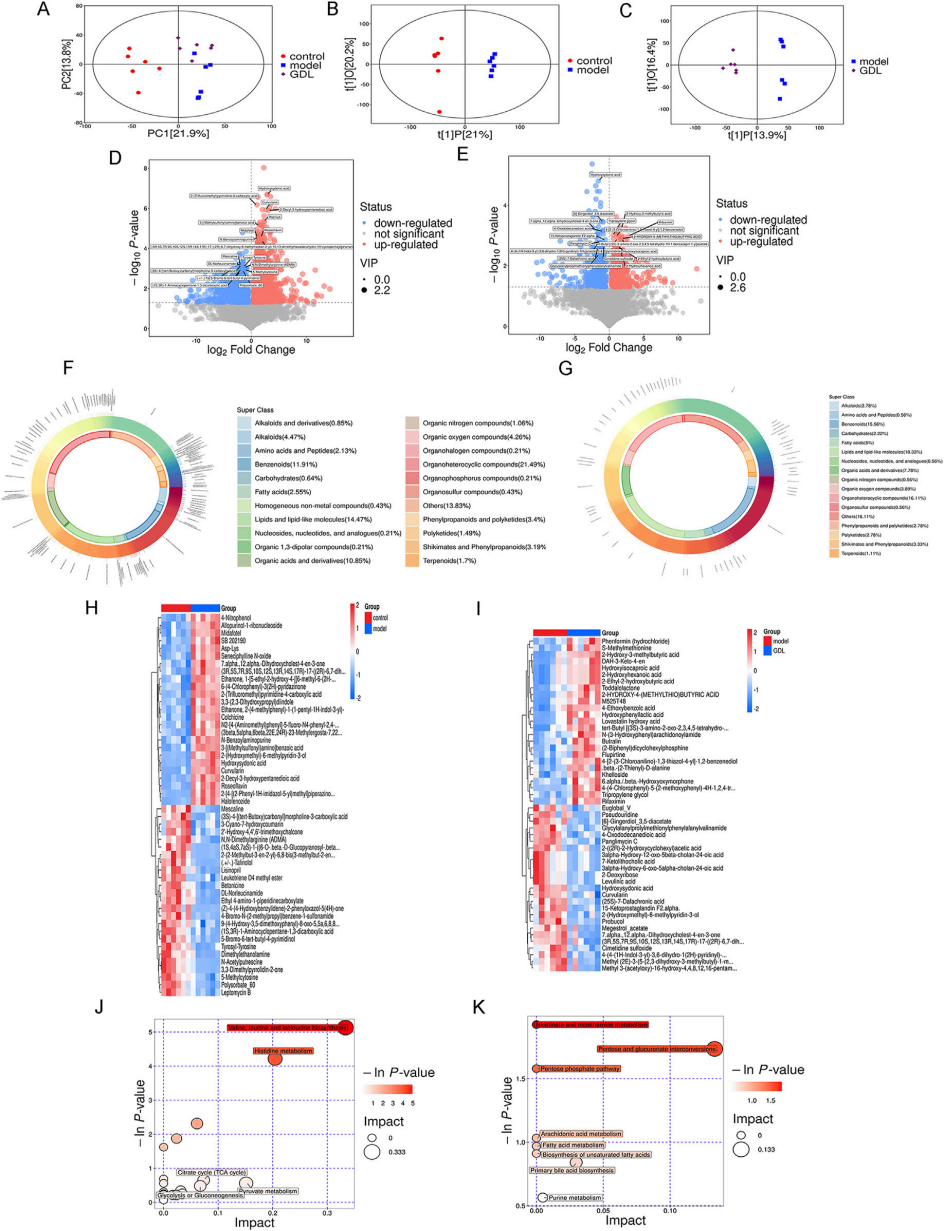
Differences in gut metabolomic profiles. Score plots of the principal component analysis (PCA) among the three groups **(A)**, Score plots of the orthogonal partial least squares discriminant analysis between the control and model groups **(B)**, as well as the model and GDL-M groups **(C)**. Volcano plots of the control and model groups **(D)**, as well as the model and GDL-M groups **(E)**, where each point represents a metabolite. Red and blue dots represent upregulated and downregulated metabolites, respectively. Proportions of differential faecal metabolite classes between the control and model groups **(F)**, as well as the model and GDL-M groups **(G)**. The heatmap of the significantly different metabolites across the control and model groups **(H)**, as well as the model and GDL-M groups **(I)**. Related pathways were screened by comparing the control and model groups **(J)**, as well as the model and GDL-M groups **(K)**.

The probable metabolites and pathways impacted by medium-dose GDL were further identified using non-targeted metabolomics. According to the principal component analysis (Figure 7A), the metabolic profiles of the GDL group showed some overlap with the model group, while the model and control group profiles were different. To ascertain the metabolic changes in the three groups, we conducted orthogonal partial least squares discriminant analysis (OPLS-DA). According to the OPLS-DA models (Figures 7B,C), the metabolomic data from the control and model groups, as well as from the model and GDL groups, significantly differed. This observation confirmed that the model group was different from the control group and that the model group metabolites were altered by the GDL intervention. Compared with the control group, as illustrated in the volcano plot, 2630 differentially significant metabolites were identified in the model group (Figure 7D), of which 1728 were increased and 902 were decreased. The majority of these differential metabolites were concentrated in organoheterocyclic compounds (21.49%), followed by lipids and lipid-like molecules (14.47%), benzenoids (11.91%), and organic acids and their derivatives (10.85%) (Figure 7F). Moreover, GDL treatments affected 1,076 differential metabolites in the TX mice (Figure 7E), of which 566 were increased and 510 were decreased. The majority of these differential metabolites were concentrated in lipids and lipid-like molecules (18.33%), with organoheterocyclic compounds (16.11%) and benzenoids (15.56%) following (Figure 7G). The reversal of metabolite levels in the corresponding clusters was assessed, and details related to the differential metabolites are listed. Furthermore, a global overview of the differential metabolic features is depicted in heatmaps (Figures 7H,I). We then analysed the metabolic pathways associated with differing metabolites using the Kyoto Encyclopedia of Gene and Genome pathway database. Valine, leucine, and isoleucine biosynthesis, histidine metabolism, citrate cycle (TCA cycle), glycolysis or gluconeogenesis, and pyruvate metabolism were the pathways that were impacted when the model and control groups were compared (Figure 7J). Nicotinate and nicotinamide metabolism, pentose and glucuronate interconversions, pentose phosphate pathway, arachidonic acid metabolism, fatty acid metabolism, biosynthesis of unsaturated fatty acids, and primary bile acid biosynthesis were the pathways that were impacted when the GDL and model groups were compared (Figure 7K).

**FIGURE 8:**
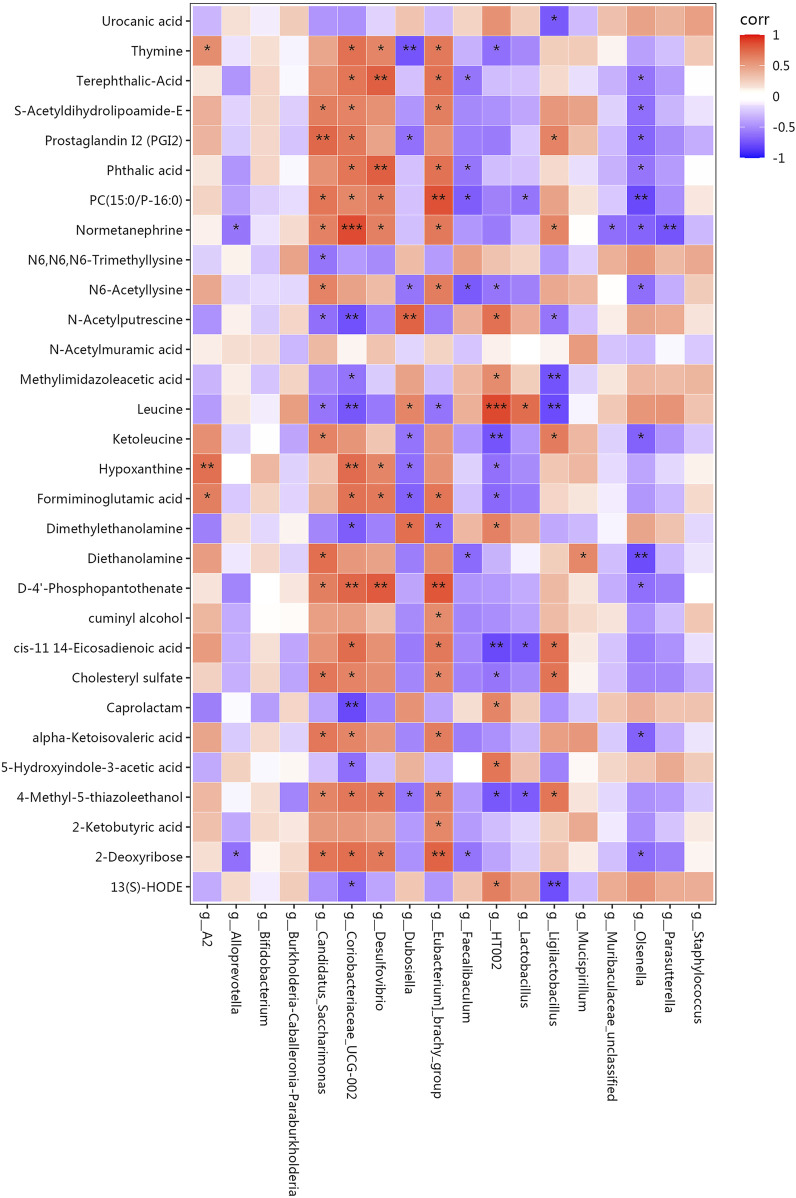
Pearson’s correlation coefficients between the abundance of crucial differentially enriched gut bacterial genera and differential metabolites. Correlation effect is represented by a colour gradient ranging from red (negative correlation) to blue (positive correlation). *p < 0.05, **p < 0.01, ***p < 0.001.

Ultimately, we employed Spearman’s correlation analysis to assess the association between the gut microbiome and metabolic products. Lipids and lipid-like molecules, such as S−acetyldihydrolipoamide−E, prostaglandin I2, and PC (15:0/P−16:0) showed a positive correlation with the abundance of *Candidatus_Saccharimonas* and *Coriobacteriaceae_UCG-002*. On the other hand, there was a negative relationship found between the abundance of *Olsenella* and these metabolic products. Amino acids and peptides, such as formiminoglutamic acid and 4-Methyl-5-thiazoleethanol, were positively correlated with the abundance of *Coriobacteriaceae_UCG−002*, *Desulfovibrio*, and *[Eubacterium]_brachy_group,* and negatively correlated with those of *Dubosiella* and *HT002*. Fatty acids, such as cis-11.14-eicosadienoic and alpha-ketoisovaleric acids, showed a positive correlation with the abundance of *Coriobacteriaceae_UCG−002* and *[Eubacterium]_brachy_group*. Organic acids and their derivatives and carbohydrates, such as, D-4′-phosphopantothenate and 2-deoxyribose, were positively correlated with the abundances of *Candidatus_Saccharimonas, Coriobacteriaceae_UCG−002*, *Desulfovibrio*, and *[Eubacterium]_brachy_group,* and negatively correlated with that of *Olsenella*. N-acetylputrescine, and leucine showed a positive correlation with the abundances of *Dubosiella* and *HT002,* and a negative correlation with those of *Candidatus_Saccharimonas, Coriobacteriaceae_UCG−002*, and *Ligilactobacillus* (Figure 8).

## Discussion

WD is a hereditary disorder characterized by abnormal copper metabolism, which can lead to psychiatric, neurological, and liver issues. The primary hepatic pathology observed in WD is liver fibrosis, which is caused by impaired copper excretion and accumulation in the liver (Bandmann et al., 2015). The copper deposited in tissues can be combined with heavy metal chelators, such as penicillamine, to form a soluble complex that is excreted in the urine (Li et al., 2016). However, the high frequency of adverse reactions during clinical treatment, along with the serious side effects of heavy metal chelators, limits their utilization. The gut microbiota, which is essential for digestion, metabolism, and nutrient absorption, is strongly linked to various liver diseases (Cesaro et al., 2011; Xu et al., 2022). WD causes changes in the gut microbiome and these imbalances influence the development of WD (Rager and Zeng, 2023). Therefore, therapies that rebalance microbiota composition and function are promising agents for ameliorating WD-induced liver damage (Albillos et al., 2020). Microbial diversity can be regulated by the interaction between TCMs and gut microbiota.

WD-associated liver fibrosis is characterised by excessive ECM accumulation, and early intervention is essential for treating WD cirrhosis and preventing the progression to liver failure (Chen et al., 2024). GDL has been shown to mitigate hepatic damage in TX mice by inhibiting the Wnt/β-catenin signaling pathway, thereby reducing inflammation and oxidative stress (Cheng et al., 2023). Furthermore, GDL shows promise in mitigating WD liver damage by preventing ferroptosis through the regulation of the HSF1/HSPB1 pathway (Zhao et al., 2024). In this study, we investigated the therapeutic impact of GDL on liver fibrosis and the mechanisms behind these effects using TX mice, which are the best model for studying WD-related liver fibrosis (Reed et al., 2018). Our research demonstrated that GDL administration significantly improved the levels of liver function biochemical indices, including AST, ALT, ALB, and TBIL; decreased the levels of liver fibres, such as HA, LN, PCⅢ, IV-C, and HYP; decreased the levels of the inflammatory factors IL-6 and IL-1β in the serum; reduced the deposition of α-SMA and collagen I in the liver; and decreased the liver coefficient. Additionally, pathological analyses demonstrated liver injury and fibrosis in the model group. The pathological alterations in the liver were lessened by GDL administration. GDL ameliorated WD liver fibrosis specific pathologies without altering body weight, reflecting its targeted efficacy.

In WD-associated liver fibrosis, decreased expression of ileal tight junction proteins, including ZO-1 and claudin-1, are linked to intestinal barrier dysfunction (Ren et al., 2014). In this study, ileal permeability was restored by GDL intervention, which increased ZO-1 and claudin-1 expression in the intestinal epithelium.

The present study confirms the impact of WD-associated liver fibrosis on altering gut microbiota and faecal metabolites. Previous studies have investigated the characteristics of the gut microbiome in WD patients, revealing a disruption in the balance of gut microbiota and a decrease in bacterial diversity (Geng et al., 2018; Cai et al., 2020; Cai et al., 2024). In contrast to the clinical research reports, we observed no noticeable alterations in the α-diversity between the control and model mice. We assume that this discrepancy could be caused by the phenotypic selection of model mice. In this study, we analysed liver damage in TX mice; however, a previous study reported no unified clinical phenotype of patients with WD. While alpha diversity quantifies overall microbial complexity, it is well-documented that dysbiosis in fibrotic liver diseases often manifests as pathobiont expansion and symbiont depletion without significant diversity changes. For example, AFLD-associated dysbiosis frequently shows unchanged Shannon index but marked Proteobacteria overgrowth (Chi et al., 2023). There was no evident alteration in the α-diversity index following GDL administration. However, β-diversity analysis revealed significant dissimilarity between the control and model groups, as well as between the model and GDL groups, consistent with the findings of the previous study.

Bacteroidetes and Firmicutes were the predominant bacteria phyla detected in all groups. Prior research has indicated a decline in the variety of the gut microbiota, with a lower abundance of Bacteroidetes and higher abundances of Firmicutes, Proteobacteria, and Fusobacteria in patients with WD than those in healthy individuals (Geng et al., 2018; Cai et al., 2024). We observed a lower abundance of Bacteroidota and higher abundances of Desulfobacterota, Patescibacteria, and Actinobacteriota in the model group. Bacteroidetes primarily produce acetate and propionate, the primary short-chain fatty acids (SCFAs) in the colon. SCFAs induce IL-22 production by innate lymphocytes and CD4 T cells, thereby promoting intestinal epithelial repair and regeneration, which provides a protective mechanism for the host. Additionally, Bacteroidetes contribute to the maintenance of gut microbial diversity and ecological balance by interacting with other microorganisms (Parada Venegas et al., 2019). The relative abundance of *Bacteroides* has been reported to be significantly low at different pathological stages of liver disease and decrease gradually with the progression of liver disease (Milosevic et al., 2019; Hsu and Schnabl, 2023). Our findings are consistent with those of previous studies showing that the relative abundance of Bacteroidetes is significantly lower in liver diseases. However, following GDL treatment, there was no change in the relative abundance of *Bacteroides*. Additionally, GDL administration predominantly decreased the abundance of Patescibacteria.

The results of this study showed changes in the abundance of several genera, including a higher abundance of *Desulfovibrio, Candidatus_Saccharimonas, A2, Bifidobacterium, Coriobacteriaceae_UCG-002, Ligilactobacillus, Parasutterella, Mucispirillum,* and *Eubacterium]_brachy_group* and a lower abundance of *Olsenella*, *HT002*, *Burkholderia-Caballeronia-Paraburkholderia*, *Faecalibaculum*, *Dubosiella, Staphylococcus, Lactobacillus, Muribaculaceae_unclassified,* and *Alloprevotella* in the model group than in the control group. GDL treatments significantly increased the relative abundance of *HT002* and decreased those of *Desulfovibrio, Coriobacteriaceae_UCG-002, A2,* and *[Eubacterium]_brachy_group*. Intestinal inflammation and epithelial permeability are promoted by *Coriobacteriaceae_UCG-002* and *Desulfovibrio* (Yu et al., 2023), which were reversed after GDL administration. *Coriobacteriaceae_UCG-002, Desulfovibrio*, and *[Eubacterium]_brachy_group* were positively correlated with AST levels, liver fibrosis, liver coefficient, and inflammation and negatively correlated with ZO-1 and ALB levels. Moreover, GDL treatment increased the abundance of *HT002*, which showed a positive correlation with intestinal barrier integrity and a negative correlation with liver function, liver fibrosis, liver coefficient, and inflammation. *Bifidobacterium* is a well-known beneficial bacterium that regulates the gut microbiota and promotes human health. Our microbiological assays showed a higher abundance of *Bifidobacterium* in the model group, in line with earlier Sprague-Dawley rat experiments (Han et al., 2010). The abundance of *Bifidobacterium* showed no changes after GDL administration. As we know, *Bifidobacterium* utilizes host-derived mucin glycans as carbon sources, gaining a competitive edge when copper restricts nutrient availability for other taxa (Li et al., 2023). The growth mechanism of *Bifidobacterium* and *Ligilactobacillus* in WD was unclear, and we speculated that copper hindered certain organisms, thus promoting a better environment for beneficial bacteria. While *Bifidobacterium* and *Ligilactobacillus* are typically anti-inflammatory, their elevation in TX mice may reflect strain-specific adaptations to copper overload and liver injury. Further studies are needed to dissect their functional duality in this context.

According to metabolomics, WD significantly changed the faecal metabolic profile, which primarily involved organoheterocyclic compounds, lipids, lipid-like molecules, benzenoids, and organic acids and their derivatives. Further analysis indicated that valine, leucine, and isoleucine biosynthesis, histidine metabolism, citrate cycle (TCA cycle), glycolysis or gluconeogenesis, and pyruvate metabolism were the affected pathways, characterised by upregulated levels of alpha-ketoisovaleric acid, 4-methyl-2-oxopentanoate, formiminoglutamic acid, and S-acetyldihydrolipoamide-E, along with downregulated levels of leucine, urocanic acid, and methylimidazoleacetic acid. After GDL administration, lipids, lipid-like molecules, organoheterocyclic compounds, and benzenoids were the primary components of the differential metabolites. GDL predominantly affected the nicotinate and nicotinamide metabolisms, pentose and glucuronate interconversion, pentose phosphate, arachidonic acid metabolism, fatty acid metabolism, biosynthesis of unsaturated fatty acids, primary bile acid biosynthesis, and purine metabolism pathways. Nicotinic acid, D-xylose, deoxyribose, prostaglandin I2, L-palmitoylcarnitine, gamma-linolenic acid, chenodeoxycholic acid-glycine conjugate, and adenine were enriched through gut microbiota-mediated metabolic processes. Among them, prostaglandin I2 exerted anti-fibrotic effects by activating receptors, increasing the intracellular cyclic adenosine monophosphate and protein kinase A activities and inhibiting fibroblast proliferation and ECM protein synthesis (Zhang et al., 2024) which was upregulated by GDL administration. GDL significantly increased the levels of L-palmitoylcarnitine, an oxidative product of fatty acids, indicating that GDL promotes the β-oxidation process of liver fatty acids and further alleviates liver steatosis. Nicotinic acid functions as a precursor in the biosynthesis of two essential coenzymes, nicotinamide adenine dinucleotide and nicotinamide adenine dinucleotide phosphate. Augmenting intrahepatic concentrations of nicotinamide adenine dinucleotide via nicotinic acid supplementation has been demonstrated to significantly improve liver function, reduce inflammatory responses, inhibit hepatocyte apoptosis, and promote the repair and regeneration of liver tissue (Skok, 2022; Pan et al., 2024). Gamma-linolenic acid is an essential component of the mitochondria and cell membranes, participates in lipid and cholesterol metabolism, and upregulated by GDL administration (Sergeant et al., 2016). While our analysis focused on above pathways, other metabolites showed significant changes. These may represent novel therapeutic targets requiring further validation.

## Conclusion

Different GDL doses were administered to TX mice, significantly ameliorating the symptoms of WD-associated liver fibrosis. This was evidenced by a decrease in liver fibrosis and inflammatory indices, an improvement in liver function indices in the serum, and a mitigation of liver histopathology and intestinal barrier damage. Prostaglandin I2, L-palmitoylcarnitine, and gamma-linolenic acid were elevated and health-associated gut bacterial genera, such as *HT002,* were enriched by a medium GDL dose. Thus, GDL shows potential anti-fibrosis therapeutic effects in TX mice by regulating the composition of gut microbiota and the production of lipid metabolites. While our multi-omics analyses revealed significant associations between GDL treatment, gut microbiome remodeling, and liver fibrosis attenuation, the causal role of specific microbial taxa or metabolites requires further validation. Future studies employing fecal microbiota transplantation (FMT) from GDL-treated mice to germ-free models, or targeted metabolite supplementation, will be essential to establish mechanistic causality.

## Data Availability

The 16S rRNA data presented in the study are deposited in the NCBI SRA (BioProject) repository, accession number PRJNA1268003. Further inquiries can be directed to the corresponding authors.
